# Heterogeneity of repetition abilities in logopenic variant primary progressive aphasia

**DOI:** 10.1590/1980-57642021dn15-030014

**Published:** 2021

**Authors:** Joel Macoir, Vicent Martel-Sauvageau, Liziane Bouvier, Robert Laforce, Laura Monetta

**Affiliations:** 1 Faculty of Medicine, Department of Rehabilitation, Laval University – Quebec, QC, Canada.; 2 CERVO, Brain Research Centre – Quebec, QC, Canada.; 3 Center for Interdisciplinary Research in Rehabilitation and Social Integration – Quebec, QC, Canada.; 4 Faculty of Medicine, Department of Medicine, Laval University – Quebec, QC, Canada.; 5 Interdisciplinary Memory Clinic, Laval University Hospital Center – Quebec, QC, Canada.; 6 Research Chair in Progressive Primary Aphasias, Lemaire Family Foundation – Quebec, QC, Canada.

**Keywords:** primary progressive aphasia, differential diagnosis, acquired language disorders, psycholinguistics, afasia primária progressiva, diagnóstico diferencial, distúrbios de linguagem adquiridos, psicolinguística

## Abstract

The differential diagnosis of primary progressive aphasia (PPA) is challenging due to overlapping clinical manifestations of the different variants of the disease. This is particularly true for the logopenic variant of PPA (lvPPA), in which such overlap was reported with regard to impairments in repetition abilities. In this study, four individuals with lvPPA underwent standard neuropsychological and language assessments. The influence of psycholinguistic variables on their performance of in word, nonword and sentence repetition tasks was also specifically explored. Some level of heterogeneity was found in cognitive functions and in language. The four participants showed impairment in sentence repetition in which their performance was negatively affected by semantic reversibility and syntactic complexity. This study supports the heterogeneity of lvPPA with respect to the cognitive and linguistic status of participants. It also shows that sentence repetition is influenced not only by length, but also by semantic reversibility and syntactic complexity, two psycholinguistic variables known to place additional demands on phonological working memory.

## RESUMO.

O diagnóstico diferencial da afasia progressiva primária (APP) é desafiador devido às sobreposições das manifestações clínicas das diferentes variantes da doença. Isso é particularmente verdadeiro para a variante logopênica do APP (APPlg), em que tal sobreposição foi relatada em relação à deficiências nas habilidades de repetição. No presente estudo, quatro indivíduos com APPlg foram submetidos à avaliações neuropsicológica e de linguagem. A influência de variáveis psicolinguísticas em seu desempenho de palavras, não-palavras e tarefas de repetição de frases também foi especificamente explorada. Certo nível de heterogeneidade foi encontrado nas funções cognitivas e na linguagem. Os quatro participantes apresentaram prejuízo na repetição de frases em que seu desempenho foi afetado negativamente pela reversibilidade semântica e complexidade sintática. O estudo apoia a heterogeneidade do APPlg no que diz respeito ao status cognitivo e linguístico dos participantes. Mostra também que a repetição de sentenças é influenciada não apenas pelo comprimento, mas também pela reversibilidade semântica e complexidade sintática, duas variáveis psicolinguísticas conhecidas por colocarem demandas adicionais na memória operacional fonológica.

## INTRODUCTION

The diagnostic criteria of neurodegenerative diseases are constantly evolving, as in the case of primary progressive aphasia (PPA). PPA is a neurodegenerative syndrome associated with atrophy of the frontal and temporal regions of the left hemisphere, typically resulting in language impairment. PPA is a heterogeneous condition, in which the most prominent clinical feature is difficulty with language (deficit of language production, object naming, syntax, or word comprehension), while other cognitive domains are not affected at onset and in the early stages of the disease.^1^ Language impairment characterization is particularly useful for the differential diagnosis of PPA. According to an international consensus group, PPA can have three distinct variants: the non-fluent/agrammatic variant (nfvPPA), the semantic variant (svPPA), and the logopenic variant (lvPPA).^2^ Although the criteria of PPA variants differ in terms of language manifestations, patterns of atrophy and underlying neuropathology (frontotemporal lobar degeneration in nfvPPA and svPPA; Alzheimer’s disease in the majority of lvPPA), differential diagnosis remains challenging due to overlapping clinical manifestations of the different profiles. Unclassifiable cases of PPA were reported in several studies in which the diagnostic criteria for PPA were used.^3^ These classification issues appeared to be particularly important for lvPPA. For example, Sajjadi et al.^4^ carried out a factor analysis of the results of 46 individuals diagnosed with PPA in a set of language tests. The results of this analysis were consistent with the existence of two variants, one typical of svPPA (semantic deficits) and the other of nfvPPA (agrammatism and apraxia of speech). However, the analysis did not identify a cluster compatible with the clinical profile of lvPPA. While the clinical profile of svPPA is relatively well-defined, there is some overlap between nfvPPA and lvPPA, particularly with regard to impairments in repetition abilities.^5^ The lvPPA, the most recently identified PPA variant,^6^ is characterized by the following key features: 1) anomia in spontaneous speech and confrontation naming, and 2) impaired repetition of sentences and phrases. Moreover, at least three of the following features must be present: production of phonological errors, preservation of semantic memory, preservation of articulation and prosody, and/or absence of agrammatism. In lvPPA, impairment in repetition abilities has been attributed to a reduced capacity of working memory resources.^7^ Working memory is essential to successfully rehearse information in sentence repetition since it is involved in linguistic information retention, as well as in the computation of syntactic structures.

However, the guidelines proposed by Gorno-Tempini et al.^2^ lack specificity regarding the tests to be used to differentiate the three PPA variants^8^ and, more particularly, lvPPA.^9^ PPA classification guidelines are also silent regarding the psycholinguistic variables to control in language tests. The objective of this study is to investigate the influence of psycholinguistic variables known to challenge working memory (length, lexicality, syntactic complexity, and semantic reversibility) on the performance of four individuals with lvPPA in word, nonword and sentence repetition tasks.

## METHODS

### Participants

**Figure 1.**
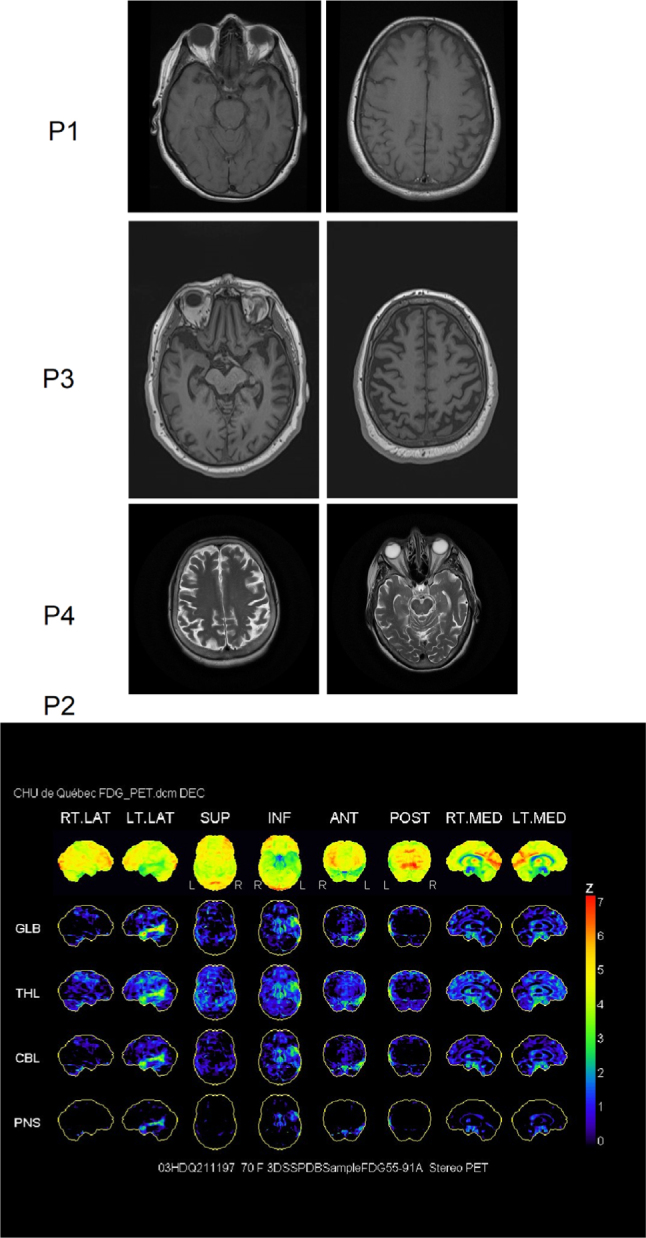
Transverse weighted MR images for P1, P3 and P4 and FDG-PET scans of P2. P1. Axial 3DT1 MRI showing left anterior temporal and left hippocampal (left image) and left parietal atrophy (right image). P3. Axial 3DT1 MRI showing left temporal (left image) and left posterior parietal atrophy (right image). P4. Axial T2 MRI showing left parietal (left image) and left temporal atrophy (right image). P2. 18F-FDG-PET showing left temporoparietal hypometabolism.

Four individuals with lvPPA (two men and two women) were recruited at the Clinique Interdisciplinaire de Mémoire du Centre Hospitalier Universitaire de Québec. They were diagnosed by an experienced neurologist using Gorno-Tempini et al.’s^2^ criteria and underwent structural brain imaging (P1, P3, P4) and metabolic brain imaging (P3) in order to confirm the probable clinical diagnosis (see Figure 1).

Twelve healthy controls (HCs) (ten females, two males), who matched the four lvPPA participants in age (lvPPA: mean=66.75, SD=8.14; HC: mean=67.08, SD=6.4; p=0.94) and years of education (lvPPA: mean=14.25, SD=5.25; HC: mean=14.42, SD=4.34; p=0.96) were recruited. All of the HC participants were in good physical and mental health, reported no significant complaints related to cognition and performed within the normal range on the Montreal Cognitive Assessment. Quebec French was the first language of all of the participants. Written informed consent to be included in the study was obtained from all lvPPA and HC participants in accordance with the latest articles of the Declaration of Helsinki. The study was approved by the Ethics Committee for Sector Research in Neurosciences and Mental Health (project 2017-164).

### Background testing

The four participants with lvPPA underwent standard neuropsychological and language testing batteries. The neuropsychological battery included tests of general cognition,^10^ general linguistic abilities,^11^ inventory of depression and neuropsychiatric symptoms,^12^ ^,^ ^13^ executive functions^14^ ^,^ ^15^ and working memory,^16^ non-verbal episodic memory,^17^ visuo-perceptual abilities,^18^ and sensorimotor execution.^19^ The language battery included tests of lexical access and semantic memory,^20^ ^-^ ^22^ reading and writing,^22^ and sentence comprehension.^23^ In all of these tests, the performance of the participants with lvPPA was compared to published normative data.

### Repetition tasks

The word and nonword repetition abilities of the four participants with lvPPA and the twelve HCs were assessed with the repetition subtests of the *batterie d’évaluation cognitive du langage* (battery of cognitive assessment of language).^22^ The word list included 15 stimuli manipulated for length (monosyllabic=5, bisyllabic=5, trisyllabic=5) and syllable complexity (simple=7, complex=8). The nonword list included ten stimuli manipulated for syllable length (monosyllabic=3, bisyllabic=4, trisyllabic=3) and syllable complexity (simple=5, complex=5). The total number of words and nonwords correctly repeated (i.e., exact repetition of the stimulus) was recorded. Immediate self-corrections and simple hesitations were scored as correct.

The *test français de répétition de phrases* (French sentence repetition test)^24^ was used to assess sentence repetition abilities. The test consists of 24 sentences in which length (12 short sentences of seven to eight syllables and 12 long sentences of 13 to 14 syllables), semantic reversibility (i.e. the noun phrase can either play a thematic role around the verb or not, e.g. *the boy is playing with the girl* vs *the boy is watering the flowers*) (reversible=12, non-reversible=12), and syntactic structure (active=16, passive=8) are manipulated. The total number of sentences correctly repeated (i.e., exact repetition of the stimulus) was recorded. Immediate self-corrections and simple hesitations were scored as correct. A qualitative analysis of errors produced in the sentence repetition task was also performed according to the following classification: a) word omission (missing word(s) in the repeated sentence); b) word repetition (word repeated inside the sentence); c) word addition (addition of words to the sentence); d) incorrect word order; e) phonological error (phoneme substitution, omission, addition, or deletion); f) semantic substitution (word substitution by semantically associated words).

## RESULTS

### Background testing

As shown in Table 1, the four participants showed impairment on tests of general cognition^10^ ^,^ ^11^ but showed no important signs of depression or neuropsychiatric symptoms.^12^ ^,^ ^13^ They also showed impaired executive functions^14^ ^,^ ^15^ (mental flexibility), and phonological short-term and working memory.^16^ Only participant 2 (P2) showed impairment of non-verbal episodic memory,^17^ while P2 and P4 had deficits in object recognition^18^ (visual perceptual abilities: judgement “real” – “not real” on pictures). In this task, both participants almost systematically judged the stimuli as “real”, suggesting impairment of inhibition and/or mental flexibility rather than associative agnosia. Finally, these two participants also showed ideomotor limb apraxia on tests of sensorimotor execution.^19^

**Table 1.** Participants’ demographic data and results on clinical and neuropsychological tests

Characteristics	P1	P2	P3	P4
Sex	F	F	M	M
Age (in years)	65	72	56	74
Education (in years)	19	14	17	7
Time post diagnosis (in years)	2	3	2	3
FDG-PET (hypometabolic zones)	Left parieto-temporal	Left inferior and posterior temporal	Left parieto-temporal	Left parieto-temporal
General cognitive and neuropsychiatric screening				
MoCA (30)	10 (Z=-7.8)^*^	16 (Z=-7.2)^*^	10 (Z=-6.9)^*^	10 (Z=-0.4)^*^
DTLA (100)	42 (Z=-6.5)^*^	57 (Z=-8.9)^*^	58 (Z=-15.9)^*^	44 (Z=-4.8)^*^
NPI-Q (36)	3	0	1	3
SBDI (39)	1	2	0	4
Executive functions, short-term and working memory				
Trail making test				
A (sec.)	149 (Z=-4.6)^*^	131 (Z=-3.8)^*^	82 (Z=-3.0)^*^	79 (Z=-1.9)^*^
B (sec.)	>180 (Z=-2.2)^*^	>180 (Z=-1.9)^*^	Invalid	Invalid
Digit span				
Forward	1 (Z=-5.2)^*^	5 (Z=0-.45)	3 (Z=-2.95)^*^	2 (Z=-3.9)^*^
Backward	1 (Z=-3.6)^*^	3 (Z=-1.58)^*^	2 (Z=-2.1)^*^	1 (Z=-3.0)^*^
Non-verbal episodic memory				
DMS-48				
Immediate recall (48)	46 (Z=-1.04)	39 (Z=-2.9)^*^	48 (Z=1.04)	45 (Z=-0.04)
Delayed recall (48)	45 (Z=-1.4)	40 (Z=-3.1)^*^	46 (Z=-1.04)	45 (Z=-0.05)
Visuo-perceptual abilities				
Length match task (30)	28 (Z=0.7)	25 (Z=-1.2)	25 (Z=-1.2)	25 (Z=-1.2)
Object decision – hard (32)	29 (Z=1.04)	18 (Z=-3.3)^*^	26 (Z=-0.07)	16 (Z=-4.1)^*^
Sensorimotor execution (BBEP)				
Pantomime production (10)	10	6^†^	10	9
Imitation of meaningless gestures (8)	7	3^†^	7	6^†^

* Signals an impaired performance according to published norms (Z score (patient’s score – mean/standard deviation [SD]) below 1.645 SD).

† Signals an impaired performance according to norms (score below the 5^th^ percentile). BBEP: Batterie Brève d’Évaluation des Praxies; BORB: Birmingham Object Recognition Battery; DMS-48: Delayed-Matching to Sample – 48 items; DTLA: Detection Test of Language Impairment in adults and the Aged; MoCA: Montreal Cognitive Assessment; NPI-Q: Neuropsychiatric Inventory Questionnaire; SBDI: Shortened Beck Depression Inventory.

As depicted in Table 2, the four lvPPA participants showed significant language impairments.^22^ All of them showed anomia in spontaneous speech and picture naming.^20^ They mainly produced phonological errors, while articulation, prosody and syntax were preserved. Semantic memory abilities were affected in two of the four participants (P2 and P4). All four participants were impaired in word writing to dictation, while nonword writing was impaired only in P1 and P4. In this latter task, their poor performance was due to difficulty maintaining the stimulus in short-term memory (e.g., omission of the last syllable). Word and nonword reading impairments were observed in two (P1, P4) and three participants (P1, P3, P4), respectively. Finally, three of the four participants (P1, P3, P4) showed impairment in sentence comprehension,^23^ regardless of syntactic complexity.

**Table 2.** Participants’ results on language tests.

Language domain	P1	P2	P3	P4
Lexical access and semantic memory				
Picture naming (TDQ-60)	29 (Z=-13.24)^*^	39 (Z=-8.73)^*^	54 (Z=-3.96)^*^	51 (Z=-1.97)^*^
Verbal fluency				
Free fluency	12 (Z=-3.17)^*^	20 (Z=-2.68)^*^	9 (Z=-3.29)^*^	32 (Z=-1.02)
Semantic fluency	1 (Z=-3.55)^*^	9 (Z=-1.55)	9 (Z=-3.41)^*^	10 (Z=-3.4)^*^
Orthographic fluency	2 (Z=-3.45)^*^	16 (Z=-2.43)^*^	7 (Z=-2.59)^*^	3 (Z=-2.31)^*^
Written word semantic matching (20) (BECLA)	19	14^†^	18	15^†^
Reading and writing				
Word reading (40)	33^†^	39	38	23^†^
Regular (20)	20	20	20	13^†^
Irregular (20)	13^†^	19	18	10^†^
Nonword reading (40)	25^†^	35	27^†^	13^†^
Word writing to dictation (20)	8^†^	10^†^	9^†^	4^†^
Regular (10)	5^†^	7^†^	7^†^	3^†^
Irregular (10)	3^†^	3^†^	2^†^	1^†^
Nonword writing (20)	3^†^	15	15	3^†^
Sentence comprehension (M-T battery)				
Sentence-to-picture matching (47)	27 (Z=-8.06)^*^	44 (Z=-0.03)	38 (Z=-3.61)^*^	31(Z=-3.64)^*^

* Signals an impaired performance according to norms (Z score (patient’s score – mean/standard deviation [SD]) below 1.645 SD).

† Signals an impaired performance according to norms (score below the 5^th^ percentile). BECLA: Batterie d’Évaluation Cognitive du Langage; M-T Battery: Batterie Montréal-Toulouse d’examen linguistique de l’aphasie; TDQ-60: Test de Dénomination d’images de Québec – 60 items.

### Repetition

As shown in Table 3, two of the four lvPPA participants (P1, P4) showed mild impairment of word repetition abilities without any influence of length or syllable complexity. Three participants (P1, P3, P4) were impaired in the nonword repetition task, also without any influence of length or syllable complexity. The performance of all four lvPPA participants was impaired in the sentence repetition task. The difference between the lvPPA participants’ individual scores on short vs long sentences differed significantly from the distribution of differences in the HC participants in P2 only. Sentence reversibility did not influence P4’s performance, but it influenced P2 and P3 in opposite directions. Finally, the difference between the individual scores of P2 and P4 on active vs passive sentences (active>passive) differed significantly from the distribution of differences in the HC participants.

**Table 3.** Participants’ results on repetition tests.

Repetition tasks	P1	P2	P3	P4	Controls
Word and nonword repetition (BECLA)					
Word repetition – total score (15)	13^***^	15	15	13^***^	14.92 (0.29)
Word repetition according to length					
One syllable (5)	4	5	5	4	4.92 (0.29)
Two syllables (5)	5	5	5	4	5 (-)
Three syllables (5)	4	5	5	5	5 (-)
Word repetition according to syllable complexity					
Simple syllable structure (7)	7	7	7	7	6.92 (0.29)
Complex syllable structure (8)	6	8	8	6	8 (-)
Nonword repetition – total score (10)	6^***^	10	8^**^	8^**^	9.67 (0.49)
Nonword repetition according to length					
One syllable (3)	2	3	2	2	2.83 (0.39)
Two syllables (4)	2^***^	4	3	4	3.83 (0.39)
Three syllables (3)	2	3	3	2	3 (-)
Nonword repetition according to syllable structure					
Simple syllable structure (5)	2	5	5	4	5 (-)
Complex syllable structure (5)	4	5	3^**^	4	4.67 (0.49)
Sentence repetition (TEFREP)					
Sentence repetition – total score (24)	2^***^	13^**^	15^**^	9^***^	22.17 (2.17)
Sentence repetition according to length					
Short sentences (12)	2^***^ ^††^	10^††^	9^*^	6^***^	11.5 (0.9)
Long sentences (12)	0^***^	3^***^	6^*^	3^***^	10.67 (1.5)
Sentence repetition according to reversibility					
Reversible sentences (12)	2^***^	5^***^ ^††^	8^*^ ^†^	5^***^	11.42 (1.16)
Non-reversible sentences (12)	0^***^	8	4^***^	4^***^	10.75 (1.22)
Sentence repetition according to syntactic structure					
Active sentences (16)	2^***^ ^††^	10^††^	8^*^	6^**^ ^†^	14.42 (2.19)
Passive sentences (8)	0^***^	3^***^	4^***^	3^***^	7.75 (.62)

The comparison of patients’ individual scores with the control sample was performed with the Crawford modified t-test or with a χ^2^ test in the cases where there was no standard deviation in controls (i.e., perfect scores).

* p<0.05

** p<0.01

*** p<0.001

The Revised Standardized Difference Test (Crawford & Garthwaite, 2005) was used to test whether the difference between the patients’ individual scores on similar tasks differed significantly from the distribution of differences in the control participants.

† *Z*_DCC_<0.05

†† *Z*_DCC_<0.01

BECLA: Batterie d’Évaluation Cognitive du Langage; TEFREP: TEst Français de Répétition de Phrases.

From a qualitative standpoint, all the four errors produced by the lvPPA participants on word stimuli consisted of phonological errors, while the eight errors produced on nonwords consisted of seven phonological errors and one lexicalisation.

**Figure 2.**
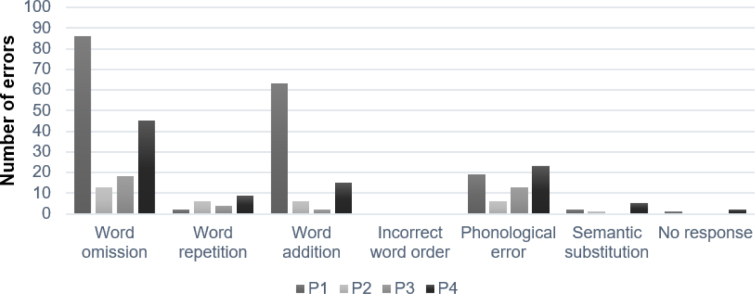
Error patterns on sentence repetition.

As shown in Figure 2, most of the errors produced by the lvPPA participants on the sentence repetition task consisted of word omissions, word additions, and word repetitions. An effect of primacy was observed, with most of these errors produced in the middle (24%) or at the end (76%) of the sentences. As proposed by Small et al. in Alzheimer’s disease,^25^ primacy effects suggest that the lvPPA participants relied more on semantic than phonological information in sentence repetition. The four participants also produced phonological errors, as well as a few semantic substitutions. Semantic substitutions are words produced in place of a content word, semantically related to the target item (e.g., *Le travailleur est accueilli par le directeur*. ‘The worker is greeted by the director’ → *Le travailleur est accueilli par le patron* ‘The worker is greeted by the boss’). The production of semantic substitutions was also observed in six out of the eight participants with lvPPA reported by Hohlbaum et al.^26^ These errors were associated with impairment of the phonological short-term memory and/or of lexical retrieval processes, rather than semantic impairment.^27^ As a whole, the error pattern on sentence repetition was very similar to that reported by Beales et al.^28^

## DISCUSSION

The four participants of the present study received a diagnosis of lvPPA according to Gorno-Tempini et al.’s criteria.^2^ Some level of heterogeneity was found in cognitive functions and in language, as reported in other studies. As expected, the four participants showed impairment in executive functions, and phonological short-term and working memory. However, impairment of episodic memory was only found in one participant, while limb apraxia was found in two participants. The impairment of non-verbal episodic memory in lvPPA was also reported in previous studies,^29^ sometimes arising from disease progression.^30^ ^,^ ^31^ Limb apraxia could also be observed in the middle stage of lvPPA.^32^ The presence of ideomotor apraxia has even shown high sensitivity for lvPPA due to Alzheimer’s pathology.^33^

The language profile largely matches Gorno-Tempini et al.’s criteria for lvPPA. All four participants presented with anomia in spontaneous speech and confrontation naming, and sentence repetition was affected. However, a deficit in semantic processing was observed in the two participants with a longer disease duration. Semantic impairment is not exceptional in lvPPA when the disease progresses^32^ ^,^ ^33^ and atrophy extends to the temporal lobe.^31^ Although written language abilities have not been studied extensively in lvPPA, we showed that word and nonword reading, as well as word and nonword spelling to dictation, may be impaired in lvPPA. In the present study, two out of the four lvPPA participants showed reading deficits. While P4’s reading performance was as impaired for regular as for irregular words, P1 was impaired for irregular words only, for which she produced phonological errors. P1 was also impaired for nonword reading, thus precluding the presence of surface dyslexia. The discrepancy between regular and irregular word reading abilities was reported in lvPPA, although to a lesser extent than in svPPA.^34^ As regards written spelling to dictation, the four lvPPA participants showed impaired performance for words, whatever their orthographic regularity level. Nonword writing was impaired only in P1 and P4, because of difficulty maintaining stimuli in short-term memory. The impairment of spelling abilities in lvPPA appears to be heterogeneous, marked with comparable difficulty in both the lexical and the sub lexical routes, and production of phonologically implausible and plausible errors.^35^ ^,^ ^36^ Finally, impairment in sentence comprehension was found in three of the four participants, regardless of syntactic complexity. Although not listed in the guidelines proposed by Gorno-Tempini et al.,^2^ sentence comprehension impairment has been repeatedly reported in studies and attributed to defective phonological short-term memory.^7^ ^,^ ^30^ ^,^ ^33^

Based on the current diagnostic criteria, repetition abilities are usually assessed with sentences in lvPPA. In the present study, we showed that repetition of single words and single nonwords may also be impaired. In this task, the reason that length did not influence performance could be due to the fact that the stimuli were not long enough to reveal the length effect expected from the impairment of phonological short-term memory. This study is also the first to show that semantic reversibility and syntactic complexity may negatively affect the performance of lvPPA participants in sentence repetition. Semantic reversibility and syntactic complexity are known to place additional demands on phonological working memory. The influence of these two variables is, therefore, not surprising, since impairment of sentence repetition abilities was attributed to reduced working memory resources in lvPPA. Leyton et al. showed a high degree of heterogeneity across lvPPA cases, probably due to extensive damage to the language network.^37^

This study has some limitations. First, it was conducted with a small number of participants, which limits its external validity. Therefore, it is crucial to replicate this study with more participants. A better control of disease duration and severity of symptoms would also be useful to confirm the relationship between the progression of the disease and the heterogeneity of cognitive manifestations. In this regard, measures of cerebral atrophy or metabolism should be added in further studies. Finally, the assessment of repetition abilities was performed using published tests with limited number of items making it difficult to precisely control the psycholinguistic variables. Further studies, using finely controlled experimental stimuli, are needed to confirm our results.

To conclude, this exploratory series of cases supports the heterogeneity of lvPPA with respect to the cognitive (i.e., impairment vs preservation of episodic memory, object recognition, sensorimotor control) and linguistic (i.e., impairment vs preservation of semantic memory, word and nonword reading, and sentence comprehension) status of participants. It also suggests that heterogeneity is present in the repetition of words and nonwords. Recently, Lukic et al. showed that the performance of lvPPA participants was poor in repeating long and non-meaningful sentences compared to short and meaningful sentences.^38^ In the present study we showed that, in addition to length and semantic plausibility, sentence repetition is influenced by semantic reversibility and syntactic complexity.
